# Heteroplasmic mitochondrial DNA variants in cardiovascular diseases

**DOI:** 10.1371/journal.pgen.1010068

**Published:** 2022-04-01

**Authors:** Claudia Calabrese, Angela Pyle, Helen Griffin, Jonathan Coxhead, Rafiqul Hussain, Peter S Braund, Linxin Li, Annette Burgess, Patricia B Munroe, Louis Little, Helen R Warren, Claudia Cabrera, Alistair Hall, Mark J Caulfield, Peter M Rothwell, Nilesh J Samani, Gavin Hudson, Patrick F. Chinnery

**Affiliations:** 1 Department of Clinical Neurosciences, School of Clinical Medicine, University of Cambridge, Cambridge Biomedical Campus, Cambridge, United Kingdom; 2 Medical Research Council Mitochondrial Biology Unit, University of Cambridge, Cambridge Biomedical Campus, Cambridge, United Kingdom; 3 Translational and Clinical Research Institute, Medical School, Newcastle University, Newcastle-upon-Tyne, United Kingdom; 4 Biosciences Institute, Newcastle University, Newcastle upon Tyne, United Kingdom; 5 Department of Cardiovascular Sciences, University of Leicester and Leicester NIHR Biomedical Research Centre, Glenfield Hospital, Leicester, United Kingdom; 6 Wolfson Centre for Prevention of Stroke and Dementia, Nuffield Department of Clinical Neurosciences, University of Oxford, Oxford, United Kingdom; 7 Clinical Pharmacology, William Harvey Research Institute, Barts and The London School of Medicine and Dentistry, Queen Mary University of London, London, United Kingdom; 8 NIHR Barts Cardiovascular Biomedical Research Centre, Barts and The London School of Medicine and Dentistry, Queen Mary University of London, London, United Kingdom; 9 Leeds Institute of Cardiovascular and Metabolic Medicine (LICAMM), University of Leeds, Leeds, United Kingdom; UNITED STATES

## Abstract

Mitochondria are implicated in the pathogenesis of cardiovascular diseases (CVDs) but the reasons for this are not well understood. Maternally-inherited population variants of mitochondrial DNA (mtDNA) which affect all mtDNA molecules (homoplasmic) are associated with cardiometabolic traits and the risk of developing cardiovascular disease. However, it is not known whether mtDNA mutations only affecting a proportion of mtDNA molecules (heteroplasmic) also play a role. To address this question, we performed a high-depth (~1000-fold) mtDNA sequencing of blood DNA in 1,399 individuals with hypertension (HTN), 1,946 with ischemic heart disease (IHD), 2,146 with ischemic stroke (IS), and 723 healthy controls. We show that the *per* individual burden of heteroplasmic single nucleotide variants (mtSNVs) increases with age. The age-effect was stronger for low-level heteroplasmies (heteroplasmic fraction, HF, 5–10%), likely reflecting acquired somatic events based on trinucleotide mutational signatures. After correcting for age and other confounders, intermediate heteroplasmies (HF 10–95%) were more common in hypertension, particularly involving non-synonymous variants altering the amino acid sequence of essential respiratory chain proteins. These findings raise the possibility that heteroplasmic mtSNVs play a role in the pathophysiology of hypertension.

## Author summary

Inherited homoplasmic variants affecting all mitochondrial DNA (mtDNA) molecules are associated with cardiovascular diseases, but the role of variants causing a mixed population of mtDNA (heteroplasmy) is not well understood. Here we sought to characterize the role of mtDNA heteroplasmic variants in hypertension, ischemic heart disease, and ischemic stroke, by deep-sequencing of the whole mtDNA molecule in 5,491 individuals including healthy controls. We measured the burden of low and intermediate level heteroplasmic single-nucleotide variants (mtSNVs) annotated in different genomic regions. In addition to an age-related accumulation of heteroplasmic mtDNA variants, we show that hypertensive individuals have a greater burden of mtSNVs variants particularly at intermediate heteroplasmy levels (HF 10–95%). These mtSNVs mostly involve non-synonymous variants in mtDNA genes coding for critical respiratory chain proteins. These findings raise the possibility that mtDNA heteroplasmy contributes to the pathogenesis of hypertension through an effect on mitochondrial respiratory chain function.

## Introduction

Mitochondrial dysfunction has been implicated in the pathogenesis of several cardiovascular diseases [1–3], but the underlying mechanisms are poorly understood. In addition to their pivotal role in oxidative metabolism and the synthesis of adenosine triphosphate (ATP), mitochondria are a potent source of reactive oxygen species, regulate intracellular calcium levels, and act as hubs for canonical cell signaling pathways implicated in several cardiovascular disorders [3]. Although the disruption of mitochondrial function could be a consequence of a primary cardiovascular disease mechanism, compelling evidence for a causal role is emerging from the genetic analysis of both the mitochondrial DNA (mtDNA) and nuclear genomes, where inherited genetic variants are associated with several cardiovascular risk factors including adipose measures, glucose/insulin levels, type 2 diabetes and cholesterol levels [4].

MtDNA codes for 13 essential respiratory chain peptides and 24 RNAs required for ATP synthesis [5]. Rare severe single nucleotide variants of mtDNA (mtSNVs) cause maternally inherited diseases that often involve the cardiovascular system, leading to cardiomyopathy and stroke-like episodes [6–9], and exacerbating cardiovascular disease risk factors including hypertension [10] and diabetes mellitus [11] through an effect on oxidative phosphorylation. These variants typically only affect a proportion of the many mtDNA molecules present within each cell (heteroplasmy). On the other hand, common maternally inherited polymorphic mtSNVs have also been associated with cardiovascular disease risk primarily linked to atherosclerosis [12,13], and typically affect every mtDNA molecule (homoplasmy).

Recent high depth mtDNA sequence has shown that, contrary to previous belief, most humans harbor a mixed population of mtDNA (heteroplasmy) which is partly inherited and partly due to somatic mtSNVs acquired during life. In any one individual, the number of mtSNVs and the level of heteroplasmy (heteroplasmy fraction, HF) tend to increase during the life course [14–16], raising the possibility that acquired heteroplasmic mtSNVs also contribute to the pathogenesis of common age-related cardiovascular disorders. To address this question, we looked for associations between aggregated individual burden of mtDNA heteroplasmy, age and three common cardiovascular diseases: hypertension (HTN), ischemic stroke (IS), and ischemic heart disease (IHD). We show an age-related accumulation of heteroplasmic mtDNA variants which is greater in individuals with hypertension than controls. The variants preferentially involve non-synonymous residues predicted to affect respiratory chain proteins. These findings raise the possibility that mtDNA heteroplasmy and oxidative phosphorylation play a role in the pathogenesis of hypertension.

## Results

### Quality control of mitochondrial DNA variants

**Fig 1:**
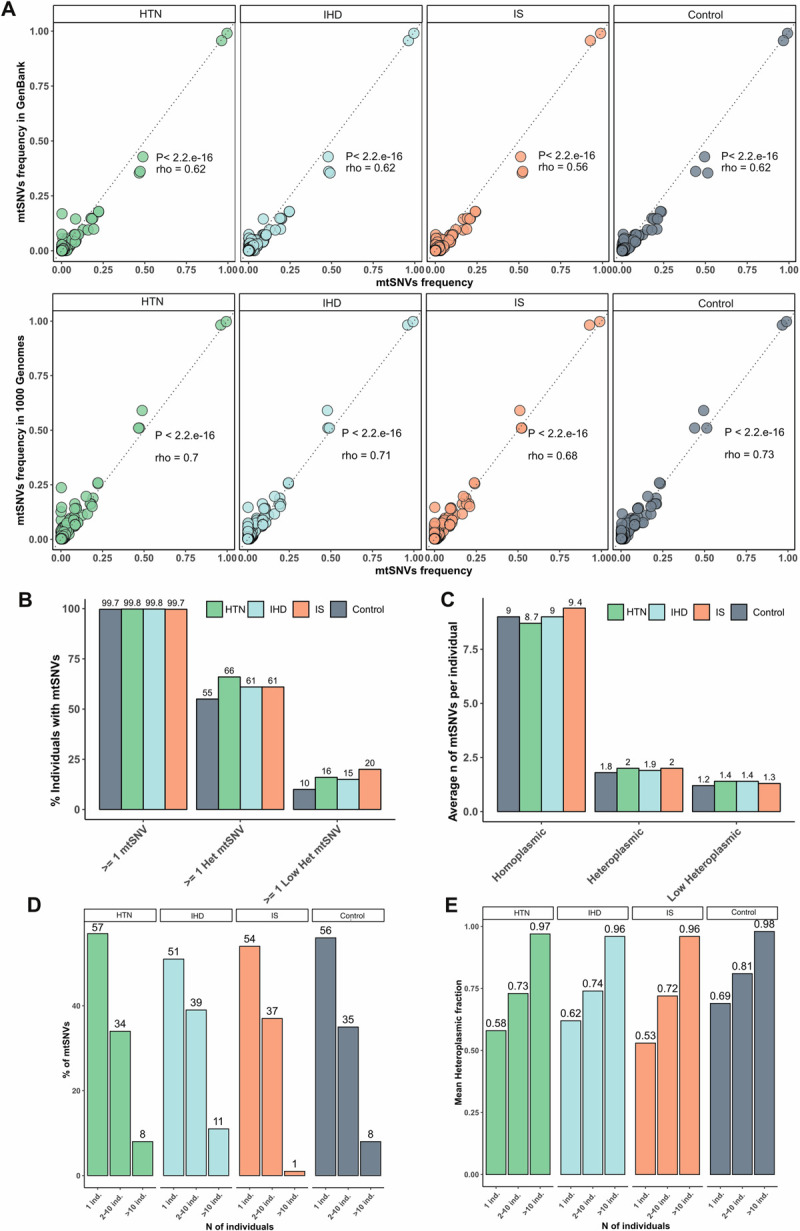
Quality control and occurrence of the mtSNVs within individuals with cardiovascular diseases and controls. A) Frequency of mtSNVs identified (HF ≥ 5%) was compared with that of two reference datasets: GenBank (top) and 1000 Genomes (bottom). Spearman rho correlation score and P-value is shown for each comparison. Frequency of variants was calculated on individuals with European mitochondrial ancestry: N = 1,338 HTN; N = 1,859 IHD; N = 2,003 IS; N = 687 controls; N = 498 1000 Genomes; N = 6,593 GenBank. B) Barplot showing the percentage of individuals within each group carrying at least one homoplasmic, intermediate heteroplasmic (Het) and low heteroplasmic (Low Het) mtSNV. C) Barplot showing the average number of homoplasmies, intermediate heteroplasmies and low heteroplasmies per individual, within each group. D) Barplot showing the percentage of mtSNVs shared by 1, 2–10 and more than 10 individuals, respectively, in each group. E) Barplot showing the average heteroplasmy of mtSNVs shared by 1, 2–10 and more than 10 individuals, respectively, in each group. HTN = Hypertension, IHD = Ischemic Heart Disease, IS = Ischemic Stroke.

We performed deep-sequencing of mtDNA derived from blood from 1,462 individuals with hypertension (HTN), 1,946 with ischemic heart disease (IHD) and 2,150 with ischemic stroke (IS) and also from 748 healthy controls. We performed variant calling of mtSNVs and applied a series of quality filters to remove putative sequencing errors and false positives (for details see also Materials and Methods). In brief: 1) we only included samples with >95% of mtDNA covered by at least one read (**S1 Appendix** and **S1 Dataset**); 2) we kept mtSNVs supported by a read depth ≥ 500x, with ≥ 25 reads carrying the alternative allele to the rCRS and present on at least one read on both strands; 3) we conservatively excluded variants with a HF<5%; 4) to remove putative wrong calls including misalignments and/or sequencing errors, we adopted the same filtering strategy described in Wei et al.[17], and removed mtDNA variants occurring in homopolymeric regions and heteroplasmic in more than 2% of the individuals in each group; 5) finally, we removed homoplasmic variants that showed more than 0.9 difference in allele frequency in the 6,593 GenBank European mtDNA genomes [18] and 498 European mtDNA genomes from the 1000 Genomes Project [19]. This reduced the number of samples with at least one filtered mtSNV to: 1,399 with HTN, 1,946 with IHD, 2,146 with IS and 723 controls (**Table A** in **S1 Appendix**). The mean sequencing read depth of the filtered samples was 1,088x (999.4x to 1178.1x; **Table A** in **S1 Appendix**) and was overall evenly distributed across the whole mtDNA molecule (**Fig A** in **S1 Appendix**), but showed a drop in the first 23 nucleotides and last 26 nucleotides of the D-loop (**S1 Dataset**), with on average 92 nucleotides per sample (corresponding to 0.5% of mtDNA) with read depth dropping to 0 (**S2 Dataset**). The drop in read depth spanning the D-loop region is explained by the short reads spanning the beginning and end of the mtDNA circular molecule, that will mostly remain unmapped. MtSNV frequencies in each study group strongly correlated with mtSNV frequencies of 6,593 GenBank [18] and of 498 1000 Genomes individuals [19] with European nuclear and mitochondrial ancestry (Spearman’s rank test P < .05; Spearman rho = 0.61 ± 0.02 sd and Spearman rho = 0.7 ± 0.02 sd, respectively **Fig 1A),** and there was no difference in the frequency (P > .05) of the major European haplogroups (H,I,J,K,U,T,V,W,X; **S2 Dataset**), between the different disease groups and controls. In total, we identified 3,156 mtDNA positions harboring at least one mtSNV with HF≥ 5% (including homoplasmies, **S3 Dataset**).

### No hotspots in the mtSNV burden in cardiovascular diseases

**Fig 2:**
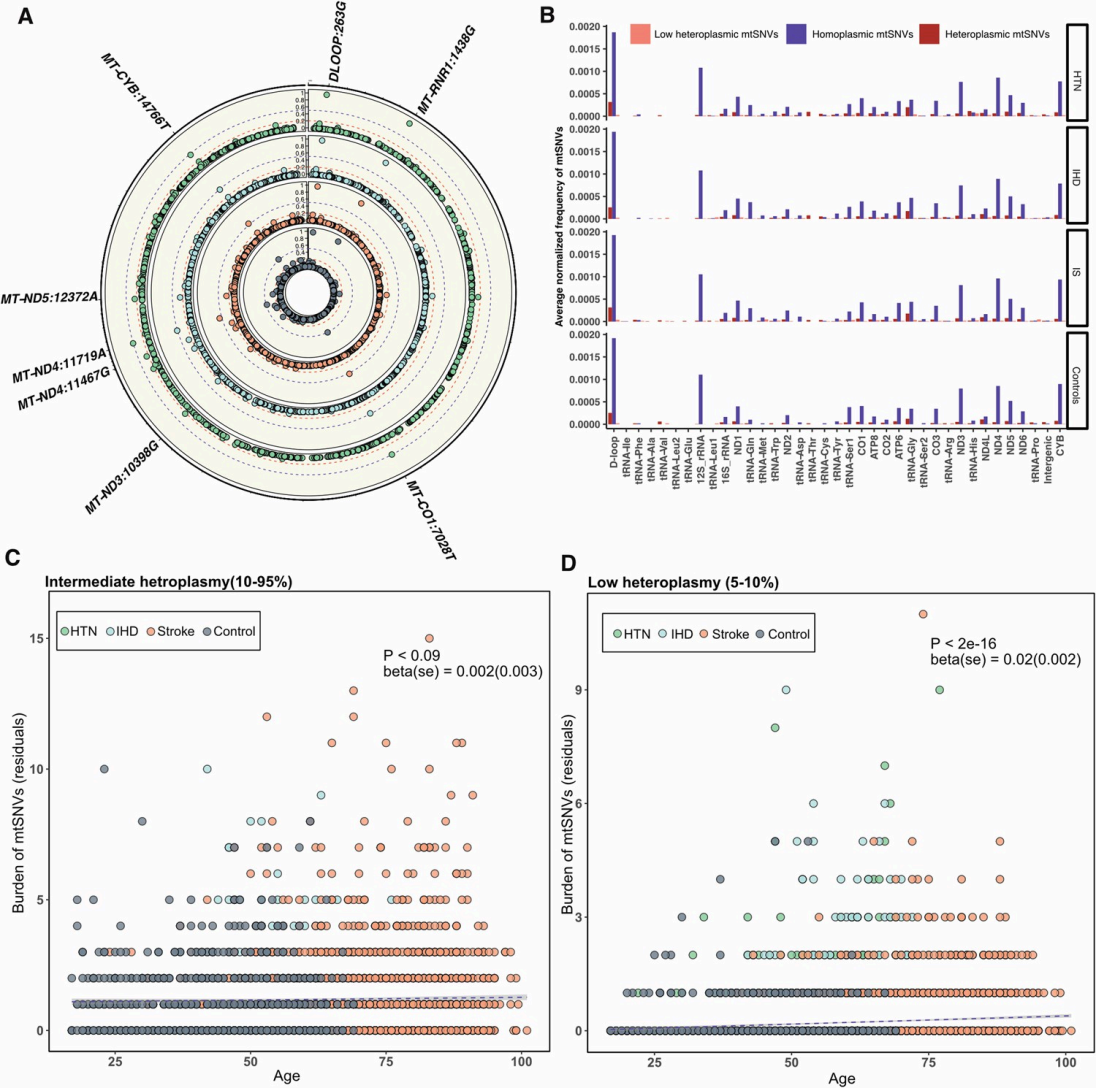
mtSNVs frequency distribution and the age-effect on individual burden of heteroplasmic mtSNVs. A) Circos plot showing the frequency of mtSNVs (HF ≥ 5%) across the whole mitochondrial genome. Red dashed line = frequency of 0.05; blue dashed line = frequency of 0.5. Common polymorphisms (indicated as mitochondrial locus: allele change) with frequency above or equal to 0.5 are labelled on the outer circle. B) Average frequency of the three classes of mtSNV (homoplasmies, intermediate and low heteroplasmies) calculated per mtDNA locus (normalized by locus length) in all the four groups. C-D) Correlation between individual burden of intermediate (HF = 10–95%) and low heteroplasmic (HF = 5–10%) mtSNVs and age. Shown on the Y axis are residuals derived from the negative binomial multivariate regression analysis with other covariates (mean read depth, gender and mitochondrial ancestry) that were used as predictors of the individual burden. P-value, beta and standard error (se) derived from the negative binomial multivariate analysis are shown for each correlation. HTN = Hypertension, IHD = Ischemic Heart Disease, IS = Ischemic Stroke.

On average, individuals harbored 10 homoplasmic and heteroplasmic mtSNVs with a HF≥ 5% (HTN mean n = 10 ± 6 sd, IHD mean n = 10 ± 6 sd, IS mean n = 11 ± 6 sd; control mean n = 10 ± 6 sd), with almost all individuals carrying at least one mtSNV (99.7% on average across all individuals, **Fig 1B**). These broke down into the following number of mtSNVs *per* individual: low heteroplasmy (5–10%), mean = 0.3 (± 0.8 sd); intermediate heteroplasmy (10–95%), mean = 1.3 (± 1.6 sd); and homoplasmic, mean 9 (± 6 sd) mtSNVs per individual (**Fig 1C**). The vast majority of mtSNVs were shared by <10 individuals, with more than a half only present in one individual (singletons) (**Fig 1D**), as seen previously [20]. As expected, homoplasmic variants were more likely to be shared by >10 individuals, whereas heteroplasmic variants (average HF = 0.6) were more likely to be rare or variants only found once in the study (singletons, **Fig 1E**). Overall, the majority of mtSNVs (97.7%) were present in <5% of individuals in each group (**Fig 2A**), with the exception of few commoner homoplasmic variants (like m.263A>G/*MT-DLOOP*, m.7028C>T/*MT-CO1*, m.11719G>A/*MT-ND4*) tagging mtDNA haplogroups, shared by more than half of the samples in each group (**Fig 2A** and **S3 Dataset)**. Similar mtSNV frequency distributions were seen across the whole mitochondrial genome and *per* gene locus, with the D-loop region and 12S rRNA being the most mutated loci across all phenotypes (**Fig 2B**). However, *per* gene locus burden test analysis for mtSNVs, performed in each heteroplasmy category, carried out by considering both annotated genome loci and overlapping ties (**Materials and Methods**), showed no significant enrichment (considering a significant Bonferroni adjusted threshold of P = 0.001 and P = 3x10^-5^, respectively). This indicates that no specific mutational hotspot could be identified in any disease group compared to controls (**S4A–S4C Dataset**).

### Age-dependent and age-independent effects on heteroplasmic mtSNVs

Multivariate regression analysis showed an age-related increase in heteroplasmic mtSNVs due to the low heteroplasmic mtSNVs (P < 2e-16, beta(se) = 0.02 (0.002)) overall (**Fig 2C and 2D** and **Table B** in **S1 Appendix)**. This trend was largely driven by HTN and IS (**Table B** in **S1 Appendix**), and only marginally by IHD (P = 0.05) and was not significant in controls (P = 0.64) (**Table B** in **S1 Appendix**), likely reflecting the proportion of older individuals (**Table A** in **S1 Appendix**). When accounting for age as a confounding covariate (**Table 1**), the HTN patients had more intermediate heteroplasmic mtSNVs than controls (P = 1.54e-05, beta(se) = 0.27(0.06)), and a marginally greater number of low heteroplasmic mtSNVs to controls (P = 0.02, beta (se) = 0.36 (0.16)), not seen for IHD or IS (**Table 1**).

**Table 1:** Effects of multiple predictors on individual heteroplasmic burden of mtSNVs.

		Intermediate heteroplasmies (HF = 10%-95%)			Low heteroplasmies (HF = 5–10%)		
Disease	Predictor	Beta	SE	*P*-value	Beta	SE	*P*-value
**HTN**	Case-Control	0.27	0.06	**1.5e-05**	0.36	0.16	0.02
	Age	0.001	0.002	0.52	0.015	0.006	*0.006
	Gender	0.08	0.05	0.13	-0.05	0.13	0.7
	Mean read depth	5e-04	1e-04	3e-04	1e-04	3e-04	0.6
	Mt ancestry	0.43	0.15	0.005	0.16	0.42	0.7
**IS**	Case-Control	0.1	0.07	0.16	0.1	0.2	0.4
	Age	0.002	0.045	0.25	0.02	0.004	4.7e-08
	Gender	0.04	0.002	2.5e-08	0.02	0.09	0.85
	Mean read depth	4.8e-04	8.6e-05	2.5e-08	5.2e-05	1.7e-04	0.8
	Mt ancestry	0.5	0.1	9.5e-07	-0.2	0.3	0.5
**IHD**	Case-Control	0.14	0.07	0.05	0.3	0.02	0.1
	Age	-0.004	0.002	0.07	0.01	0.006	0.08
	Gender	0.04	0.05	0.43	-0.16	0.13	0.2
	Mean read depth	4e-04	9e-05	3.19e-07	2e-04	2e-04	0.24
	Mt ancestry	0.65	0.2	2e-04	-0.18	0.6	0.8

Negative binomial regression analysis results regression with individual burdens of mtSNVs are shown, with effect sizes (Beta) and standard error (SE), as well as P-values of associations of the predictors used. In bold is the nominal P-value of association below the Bonferroni P-value cutoff (P = 0.008) for multiple test correction (considering alpha = 0.05 and 6 tests), denoting an association between intermediate heteroplasmy burden and HTN samples. Results are from a multivariate analysis where a dummy variable indicating case/controls was used as independent predictor together with age, gender, mean read depth and mitochondrial ancestry. HTN = Hypertension, IS = Ischemic Stroke, IHD = Ischemic Heart Disease. Mt ancestry = mitochondrial ancestry (i.e. European, African, Asian).

The greater number of intermediate heteroplasmies was largely driven by non-synonymous mtSNVs (P = 0.0006, beta (se) = 0.28 (0.08), **Table C** in **S1 Appendix**), compared to synonymous, tRNA, rRNA and the D-loop. Notably, none of the other variables available for HTN individuals (diastolic and systolic blood pressure, and smoking status) correlated with the individual burden of intermediate or low heteroplasmies (P > .02; **Table D** in **S1 Appendix**).

### Predicted functional consequences of the heteroplasmic mtSNVs

**Fig 3:**
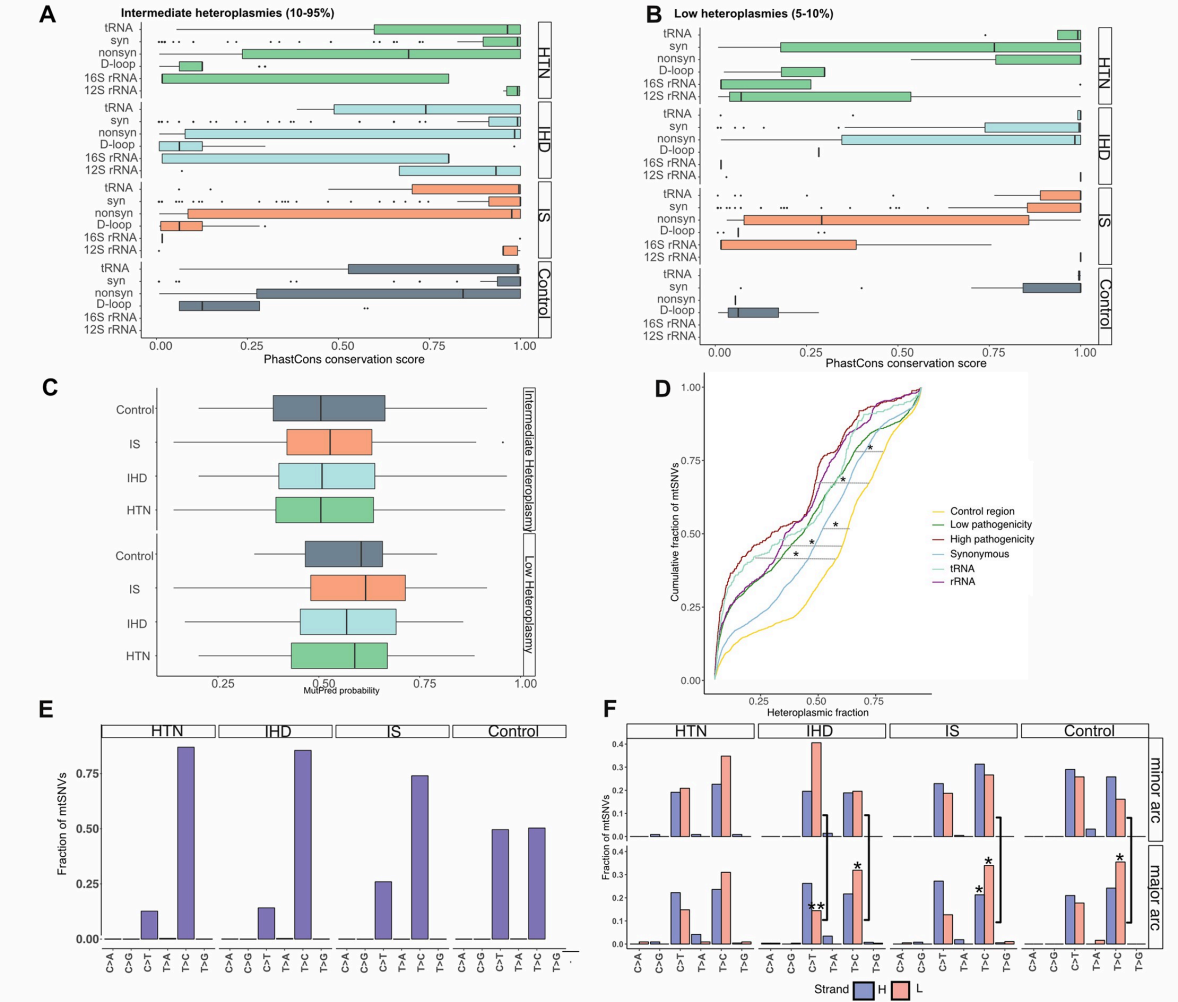
Pathogenicity of heteroplasmic mtSNVs and mitochondrial genomic signatures. A) and B) Boxplots showing the distribution of PhastCons conservation scores of intermediate and low heteroplasmic alleles in each group, respectively. Scores were calculated for six functional annotations: tRNA, 12S rRNA, 16s RNA, D-loop, non-synonymous (nonsyn) and synonymous (syn) variants. C) Boxplot showing the distribution of MuPred pathogenicity scores in intermediate and low heteroplasmic non-synonymous mtSNVs. Lower and upper hinges of the boxplots (A, B, C) in correspond to the first and third quartile of the distribution, with median in the center and whiskers spanning no further than 1.5*interquartile range. D) Cumulative fraction of heteroplasmic mtSNVs (HF = 5%-95%) across all groups. The D-loop distribution was used as reference for the Kolmogorov-Smirnov test. Stars indicate comparisons where the distribution of heteroplasmies is significantly different from the reference, according to a Bonferroni corrected p-value threshold = 0.01 (considering alpha = 0.05 and 5 tests). E) Aggregate fractions of heteroplasmic (HF = 5–95%) nucleotide changes calculated in 96 nucleotide triplets described by Alexandrov and colleagues [27], across the whole mtDNA. Each of the 6 nucleotide changes (transitions and transversions) represents an aggregated value of 16 triplets, carrying that nucleotide change at the second position of the trinucleotide. Per-nucleotide change values have been summed up altogether. The Y axis shows the frequency of mtSNVs per nucleotide change. F) Barplots show the aggregated frequencies of the 6 low heteroplasmic (HF = 5–10%) nucleotide changes that can be found in the 96 nucleotide triplets (each nucleotide change represents an aggregated value of 16 triplets), calculated within the minor arc (top) and major arc (bottom) of the mtDNA. Arcs are delimited by position m.191 (OriH) and position m.5799 (OriL), as indicated in the MITOMAP database [46]. Fractions were calculated separately for the heavy (H) and light (L) strands of the mtDNA. ** indicates two-proportions Z test P-value < 0.003 (significance threshold accounting for multiple tests), while * indicates two-proportions Z test P-value < 0.05 (no multiple tests correction). HTN = Hypertension, IHD = Ischemic Heart Disease, IS = Ischemic Stroke.

We next looked at nucleotide conservation scores across the whole mtDNA (PhastCons scores [21]) and pathogenicity scores (MutPred [22]) of non-synonymous protein-coding heteroplasmic variants (**S3 Dataset**). We found no significant difference between the disease and control groups either among conservation scores (Mann-Whitney U test P >0.05) (**Fig 3A and 3B**) or pathogenicity scores (two-way ANOVA P >.05) (**Fig 3C**). The D-loop showed the lowest mean PhastCons conservation scores in all groups (HTN mean score = 0.14 ±0.1 sd, IHD mean score = 0.1 ±0.1 sd, IS mean score = 0.09 ±0.09 sd, control mean score = 0.16 ±0.1 sd) consistent with the known high substitution rate. For the non-synonymous mtSNVs, low heteroplasmies showed a higher pathogenicity level than higher heteroplasmies (mean MutPred score = 0.58 ± 0.16 sd and score = 0.52 ± 0.17, respectively and **Fig B** in **S1 Appendix**), implying negative selection acting against non-synonymous mtSNVs during life, demonstrated by more deleterious functional consequences of lower level heteroplasmies.

We then looked at the cumulative frequency of variants in five functional groups, classifying non-synonymous changes into high and low pathogenic based on a MutPred threshold of 0.7 [23] (**S3 Dataset**). Across all groups, the non-coding D-loop tended to have a higher HF (mean HF = 0.57 ± 0.26 sd across all variants) than all other types of nucleotide changes (P < .01, two-tail Kolmogorov-Smirnoff test), followed by synonymous variants (mean HF = 0.49 ± 0.26 sd across all variants) (**Fig 3D**). Conversely, high pathogenic non-synonymous variants had the lowest heteroplasmy levels overall (mean HF = 0.34 ± 0.26 sd across all mtSNVs), as seen previously [24].

### Mitochondrial mutational signatures link low heteroplasmies to somatic events occurred during replication

Finally, we determined mtDNA mutational signatures which are known to provide insight into the underlying mechanisms of mutagenesis on both the light (L) and heavy (H)-strands of mtDNA [25,26,27]. This confirmed previous findings that C>T and T>C transitions predominate in mtDNA (99.8% of all heteroplasmic mutations found in each group) (**Fig 3E**). We also checked whether this pattern is consistent for intermediate and low-level heteroplasmies for each mtDNA strand (**Fig C** in **S1 Appendix**). This analysis revealed a H strand-asymmetry for C>T changes and L strand asymmetry for T>C changes in low heteroplasmic transitions (**Fig C** in **S1 Appendix**). This was more prominent in the mitochondrial “major arc” (defined by the origins of heavy (OriH) and light (OirL) strand replication), compared to the “minor arc” (one-tail two-proportions Z test P-value < 0.003, **S5 Dataset** and **Fig 3F**), which is consistent with a mutational mechanism linked to replication of the mtDNA molecule [25,26,28].

## Discussion

Mitochondrial dysfunction has been implicated in the pathogenesis of a variety of cardiovascular diseases (CVDs), including myocardial infarction, cardiomyopathies, some forms of arrhythmia, hypertension, atherosclerosis, and ischaemic stroke [29]. Evidence of a causal role largely comes from genetic association studies which have mainly focussed on common inherited homoplasmic variants, such as a recent study reporting D-loop homoplasmic variants associated with either reduced or increased susceptibility to CVDs, depending on the type of mutation [12]. The same study found an increased burden of heteroplasmic variants in the D-loop of controls compared to MI samples, implying a protective role of mtDNA heteroplasmies. However, this study had limited power to detect more subtle changes in heteroplasmy, and did not comprehensively study the entire mtDNA molecule. To address this, we deep-sequenced mtDNA from blood of 5,491 individuals with CVDs including hypertension, ischemic stroke and ischemic heart disease and 723 controls. The CVDs and controls analyzed were representative of the European population, and overall, showed the known increase in heteroplasmies with age [16,30], but not the previously reported association with smoking [30], despite the larger size of our study.

Accounting for available covariates including age and site-specific sequencing depth in our regression model, we found that hypertensive individuals have a greater burden of mtSNVs variants, particularly at intermediate heteroplasmy levels and affecting non-synonymous variants in genes coding for critical respiratory chain proteins required for ATP synthesis. There was no significant difference in the per-base mtDNA coverage between HTN cases and controls (Wilcoxon P-value = 0.55, **Fig D** in **S1 Appendix**), making it unlikely that variable coverage accounted for our findings. Although it is conceivable that an increased mutational burden is a consequence of hypertension, if it were a consequence of the disorder, it would equally affect synonymous and non-synonymous sites. However, the enrichment for non-synonymous variants that we observed in HTN raises the possibility that they are contributing to the pathogenesis of hypertension. This could be through an effect on mitochondrial mass or biogenesis or as consequences of impaired oxidative phosphorylation including the generation of reactive oxygen species. In keeping with this, mtDNA heteroplasmy has been associated with late onset cardiometabolic phenotypes in mice [31], adding further weight to a causal role. These findings are consistent with our analysis of the conservation scores and pathogenicity (**Fig 3**) of the intermediate heteroplasmies, where we saw no difference between HTN and the other groups. This indicates that the specific variants enriched in HTN are not qualitatively different to those seen in other diseases or healthy control participants, rather it is the burden of intermediate heteroplasmic variants that is increased in HTN.

Although it is possible that some of the specific variants we detected are due to Nuclear-mitochondrial sequences (Numts), our main conclusions are unlikely to be due to Numts for the following reasons. First, we performed sequence alignments using MtoolBox [32]. MtoolBox is effective at removing Numts by aligning reads to both the nuclear reference genome and the mtDNA reference genome, and then removing ambiguous reads [33,34]. Second, we removed all variants present at >2% frequency in the entire dataset, thus removing any common Numt contaminants. Third, our burden test analysis failed to objectively identify any recurring clusters of variants across the mtDNA. Finally, it is difficult to explain how Numts would specifically affect HTN only, and only the intermediate (10–95%) level heteroplasmies, given that overall Numt contamination is more likely to affect low-level heteroplasmies.

To provide further reassurance we repeated our analysis after removing specific variants which had similar heteroplasmy values in multiple individuals, as would be expected if variants were ‘pseudo-heteroplasmies’ due to NUMT contamination in the HTN data. To do this objectively, we calculated the coefficient of variation (CV) for the HF at each variant site (**Fig E** in **S1 Appendix**), then took a conservative approach by removing all variant sites with a CV within the bottom 25^th^ Centile (214 positions from 3165). We then repeated the HTN analysis which underpinned our main finding. Despite removing ~7% of the dataset, we still observed a robust association between intermediate level heteroplasmies and HTN (p < 2 x 10^−16^). We conclude that NUMT contamination is highly unlikely to explain our findings.

Our analysis of trinucleotide mutational signatures showed a mtDNA strand-specific pattern, particularly evident in low heteroplasmic mtSNVs (HF = 5–10%). The same signature has been previously observed with somatic mtSNVs in cancer [26,28] and is in keeping with a mtDNA replication dependent mechanism [25,26,28]. The major model for mtDNA replication is strand-asymmetric, whereby the ‘major arc’ of the molecule is exposed as a single strand before complementary strand DNA synthesis begins [35,36]. In keeping with this, the strand-bias we saw for low heteroplasmic mtSNVs was more pronounced in the major arc. Importantly, we saw the same patterns across all disease groups and controls. This supports the hypothesis that the mutation events themselves are not disease specific, but when they occur, they predispose individuals to HTN. Although we saw an association between low level heteroplasmies (5–10%) and HTN, the effect was stronger for intermediate level heteroplasmies (10–95%), consistent with a causal role analogous to a ‘dose-response’ effect.

There is emerging evidence that mtDNA and the nuclear genome interact, potentially contributing to cardiovascular disease risk [31], so it will be important to extend our findings to the nuclear genome in a larger study to provide independent replication of our findings. The detailed analysis of heteroplasmy in different functional regions of mtDNA will provide the greatest mechanistic insight and substantiate the causal role for specific non-synonymous variants that we did not have the power to detect here. Understanding how the heteroplasmies contribute to the pathogenesis of HTN will open up new opportunities for treatment development.

## Materials and methods

### Study participants

We studied n = 1,462 individuals with hypertension (HTN) from the BRIGHT study (http://www.brightstudy.ac.uk/); 1,946 with ischemic heart disease (IHD) from the BHF Family Heart Study study [37]; and 2,150 with ischemic stroke (IS) from the Oxford Vascular Study (OXVASC) study [38] and compared them to 748 healthy blood donor controls from the Wellcome Trust Case Control Consortium study (WTCCC) [39]. The participants were diagnosed using established criteria. In addition to the age and gender, systolic and diastolic blood pressure (mmHg) was available for 1,389 HTN individuals, and smoking status for 1,078 HTN individuals. One value of blood pressure per individual was available at the time of recruitment of HTN individuals in the BRIGHT study. According to the BRIGHT inclusion criteria, values of 150/100 mm Hg or higher were based on one reading, while 145/95 mm Hg or higher were a mean of three readings [40]. Some of the HTN individuals were under anti-hypertensive treatment, thus their lower blood pressure values reflect the treatment (**S2 Dataset**).

### Mitochondrial DNA sequencing

DNA was extracted from whole blood. High-depth whole mtDNA sequencing was performed using Illumina short-read sequencing on an Illumina Hiseq 2000. Fluidigm Access Array technology was used to generate ~100 tagged and indexed sub-amplicons, each of 150-200bp to allow short-read sequencing (Source Bioscience). These were tagged with sample-specific barcodes and Illumina adaptor sequences to allow a pooled library preparation. The resulting PCR products were checked for quality using the Agilent 2100 Bioanalyzer and then pooled together in equal volumes. The PCR product library was purified using AMPure XP beads and quantified with PicoGreen prior to loading for Illumina sequencing.

### Bioinformatic analysis

Sequencing reads were mapped to the human reference genome (hg19 and rCRS NC_012920.1) with the two-mapping step protocol integrated in the MToolBox pipeline [32] to exclude possible Nuclear-mitochondrial sequences (NumtS). We applied a series of quality filters to control for false positives and only included samples with >95% of mtDNA covered by at least one read (**Table A** in **S1 Appendix** and **S1 Dataset**). We analyzed mtSNVs supported by a read depth ≥ 500x, with ≥ 25 reads carrying the alternative allele to the rCRS on both strands, conservatively excluding variants with a HF<5%. Stringent filters were applied to remove putative wrong calls including misalignments and/or sequencing errors. We removed mtDNA variants occurring in homopolymeric regions and heteroplasmic in more than 2% of the individuals in each group [17]. We also removed homoplasmic variants that showed more than 0.9 difference in allele frequency in the 6,593 GenBank European mtDNA genomes [18] and 498 European mtDNA genomes from the 1000 Genomes Project [19]. These filters removed 101 positions harboring heteroplasmic variants and four positions harboring homoplasmic variants. As previously [20], we classified mtSNVs into three groups, based on their heteroplasmic fraction (HF): low-level heteroplasmies (HF = 5–10%); intermediate-level heteroplasmies (HF = 10–95%); and homoplasmies (HF > 95%). Haplogroups were assigned based on the homoplasmic variants (HF ≥ 95%) using Haplogrep2 [41] (**S2 Dataset**). PhastCons conservation scores and annotations about the mitochondrial loci of each mtSNVs were retrieved with HmtVar [42] (November 2019 update). High and low pathogenic non-synonymous variants were defined according to a MutPred score ≥ 0.7 or < 0.7, respectively, as previously described [22].

### Statistical analysis

The number of intermediate and low heteroplasmies *per* individual was used as dependent variable in a negative binomial regression analysis to assess the additive effect of each predictor on changes in individual burden of heteroplasmies, with age, sex, mean read depth, and mitochondrial ancestry as covariates (**S2 Dataset**). Systolic and diastolic blood pressure and smoking status were included as additional cofactors in the HTN analysis when possible (**S2 Dataset**). Negative binomial regression was also used to look for changes in individual heteroplasmic burden in pairwise comparisons between diseases and controls using the same covariates. *Per* locus burden analysis was performed using SKAT-O within the R package “SKAT”[43], also incorporating the same covariates, and considering: 1) mtDNA annotated genome loci; and, 2) overlapping tiles of 100bp, with an overlapping window of 10bp (**S4 Dataset**). In case 1) we looked at 37 genes encoded by the mtDNA, the non-coding D (displacement)-loop and intergenic regions defined as an aggregate region of 89 positions placed between mitochondrial gene annotations. In case 2) we divided the mtDNA reference sequence in 1,657 overlapping tiles of 100bp using the bedtools “makewindows” function [44]. The burden test was performed separately on intermediate and low heteroplasmic mtSNVs. Regression analysis and all other statistical tests stated in results were performed in R [45]. We adopted a Bonferroni adjusted P-value threshold, considering an alpha level = 0.05 and the number of tests performed in each reported comparison. Further details of the association and SKAT-O analyses performed can be found in the **S1 Appendix**.

## Supporting information

S1 Appendix **Fig A** in **S1 Appendix. Distribution of mean read depths per mtDNA position.** Mean site-specific coverage calculated across all samples that passed quality filtering and have at least 1 mtSNV (N = 6,214, reported in **S2 Dataset**). Black dots correspond to mean read depth values, connected by solid back lines. Orange lines indicate standard deviation from the mean read depth. **Fig B** in **S1 Appendix. Correlation between non-synonymous heteroplasmic fractions and MutPred probability.** Shown are Spearman’s P-value and rho coefficient of correlation. The regression line is depicted in blue with its 95% confidence interval (grey shade around the dashed line). **Fig C** in **S1 Appendix. *Per*-strand mitochondrial genomic signatures of intermediate heteroplasmies in CVDs and controls.** The barplots show the aggregated frequencies of nucleotide changes (transitions and transversions) of intermediate heteroplasmic mtSNVs (HF = 10–95%) falling within the 96 nucleotide triplettes and calculated across the whole mtDNA, in each group. HTN = Hypertension, IHD = Ischemic Heart Disease, IS = Ischemic Stroke. **Fig D** in **S1 Appendix. Distribution of mean read depths per mtDNA position in hypertension cases and controls**. Mean site-specific coverage for the hypertension samples and controls (WTCCC) which passed quality filtering and have at least 1 mtSNV. There was no significant difference in the per-base coverage between these two groups (Wilcoxon P-value = 0.55). **Fig E** in **S1 Appendix. Frequency distribution of coefficient of variation (CV) of HF values for mtDNA variants in the hypertension (HTN) cases.** CV was calculated as the ratio between standard deviation of HF and the mean HF values calculated at each position across all samples of this study (N = 6,214 samples). **Table A** in **S1 Appendix. Demographics of the three CVDs and controls.** Number of individuals reported are those with at least 1 mtSNV. HTN = Hypertension, IHD = Ischemic Heart Disease, IS = Ischemic Stroke. **Table B** in **S1 Appendix. Results of the multivariate regression analysis with heteroplasmy burden and predictors in disease/controls comparisons.** In bold are P-values below the Bonferroni corrected threshold (P < 0.008, considering alpha level = 0.05 and six tests). Mt ancestry = mitochondrial ancestry (i.e. European, African, Asian); se = standard error. **Table C** in **S1 Appendix. Results of the multivariate regression analysis with burden of intermediate heteroplasmies in Hypertension/controls comparison, *per* functional category.** In bold are P-values below the Bonferroni corrected threshold (P < 0.01, considering alpha level = 0.05 and five tests). Mt ancestry = mitochondrial ancestry (i.e. European, African, Asian); se = standard error. **Table D** in **S1 Appendix. Results of the multivariate regression analysis with heteroplasmy burden and additional predictors in Hypertension.** Results are based on N = 1,071 hypertensive individuals with non-missing predictors. In bold is the P-value below the Bonferroni corrected threshold (P < 0.02, considering alpha level = 0.05 and two tests). Mt ancestry = mitochondrial ancestry (i.e. European, African, Asian); se = standard error; SBP = systolic blood pressure; DBP = diastolic blood pressure; Smoker = ever smoked cigarettes, cigars or pipes.
(DOCX)

S1 Dataset mtDNA coverage, mean and modal read depth. Mean read depth, standard deviation, and mode values per mtDNA position, calculated across all samples that passed quality filtering and have at least 1 mtSNV (N = 6,214, reported in **S2 Dataset**).
(XLSX)

S2 Dataset List of samples and covariates. The table lists the samples that passed the quality filtering and that were used for association analyses. Age at blood collection, gender, percentage of mtDNA covered by at least one read and number of nucleotides with read depth = 0, mean sequencing read depth, haplogroup and macro-haplogroup prediction, mitochondrial ancestry are reported. Systolic and diastolic blood pressure and smoking status are available only for hypertensive individuals.
(XLSX)

S3 Dataset List of mtSNVs found. The table lists the mtSNVs found and that passed the quality filtering. The position on the rCRS reference sequence, together with reference and alternative allele are reported. Heteroplasmy classes are defined as follows: HOMO = homoplasmy (HF >95%), HETERO = intermediate heteroplasmy (HF = 10–95%), LOW.HETERO = low heteroplasmy (HF = 5–10%). MutPred predictions and probabilities, and Phastcons 100 Way scores are also reported.
(XLSX)

S4 Dataset Results of the *per* gene locus and per tile burden test using SKAT-O. The table reports the P-values of the SKAT-O burden test *per* gene locus harboring at least one heteroplasmic mtSNV. Intermediate and low heteroplasmic mtSNVs were tested separately. A: List of annotations used to calculate the burden; B: Results of the SKAT-O test using functional genomic loci; C: Results of the SKAT-O test using overlapping tiles.
(XLSX)

S5 Dataset Results of the two-proportions Z test for mtDNA mutational signatures. The table reports the P-values and test statistics of the one-tail two-proportions Z test performed between the major and minor mtDNA arcs, comparing the nucleotide substitution counts per-strand in the two arcs. P-values are reported for each nucleotide substitution, for each mtDNA strand and CVD phenotype/control group analyzed. In bold black are P < 0.05 (without taking into account multiple test comparisons), while P-values in bold red indicate P < 0.003 (taking into account multiple test comparisons).
(XLSX)
