# Supplementary material for: Heteroplasmic mitochondrial DNA variants in cardiovascular diseases
Source: PLoS Genet. 2022 Apr 1;18(4):e1010068. doi: 10.1371/journal.pgen.1010068 (PMC9007378; doi:10.1371/journal.pgen.1010068)
Supplement: S1 Appendix — Fig A in S1 Appendix. Distribution of mean read depths per mtDNA position. Mean site-specific coverage calculated across all samples that passed quality filtering and have at least 1 mtSNV (N = 6,214, reported in S2 Dataset). Black dots correspond to mean read depth values, connected by solid back lines. Orange lines indicate standard deviation from the mean read depth. Fig B in S1 Appendix. Correlation between non-synonymous heteroplasmic fractions and MutPred probability. Shown are Spearman’s P-value and rho coefficient of correlation. The regression line is depicted in blue with its 95% confidence interval (grey shade around the dashed line). Fig C in S1 Appendix. Per-strand mitochondrial genomic signatures of intermediate heteroplasmies in CVDs and controls. The barplots show the aggregated frequencies of nucleotide changes (transitions and transversions) of intermediate heteroplasmic mtSNVs (HF = 10–95%) falling within the 96 nucleotide triplettes and calculated across the whole mtDNA, in each group. HTN = Hypertension, IHD = Ischemic Heart Disease, IS = Ischemic Stroke. Fig D in S1 Appendix. Distribution of mean read depths per mtDNA position in hypertension cases and controls. Mean site-specific coverage for the hypertension samples and controls (WTCCC) which passed quality filtering and have at least 1 mtSNV. There was no significant difference in the per-base coverage between these two groups (Wilcoxon P-value = 0.55). Fig E in S1 Appendix. Frequency distribution of coefficient of variation (CV) of HF values for mtDNA variants in the hypertension (HTN) cases. CV was calculated as the ratio between standard deviation of HF and the mean HF values calculated at each position across all samples of this study (N = 6,214 samples). Table A in S1 Appendix. Demographics of the three CVDs and controls. Number of individuals reported are those with at least 1 mtSNV. HTN = Hypertension, IHD = Ischemic Heart Disease, IS = Ischemic Stroke. Table B in S1 Appendix. Results [file pgen.1010068.s001.docx]

# **Supporting Information**

**Heteroplasmic mitochondrial DNA variants in cardiovascular diseases**

Claudia Calabrese^1,2^, Angela Pyle^3^, Helen Griffin^3^, Jonathan Coxhead^4^, Rafiqul Hussain^4^, Peter S Braund^5^, Linxin Li^6^, Annette Burgess^6^, Patricia B Munroe^7,8^, Luis Little^7,8^, Helen R Warren^7,8^, Claudia Cabrera^7,8^, Alistair Hall^9^, Mark J Caulfield^7,8^, Peter M Rothwell^6^, Nilesh J Samani^5^, Gavin Hudson^3^, Patrick F. Chinnery^1,2*^

*Correspondence should be addressed to Patrick F. Chinnery ([pfc25@cam.ac.uk](mailto:pfc25@cam.ac.uk))

# **Supporting Figures and Table Legends**

**Fig A. Distribution of mean read depths per mtDNA position.** Mean site-specific coverage calculated across all samples that passed quality filtering and have at least 1 mtSNV (N = 6,214, reported in S2 Dataset). Black dots correspond to mean read depth values, connected by solid back lines. Orange lines indicate standard deviation from the mean read depth.

**Fig B. Correlation between non-synonymous heteroplasmic fractions and MutPred probability.** Shown are Spearman’s P-value and rho coefficient of correlation. The regression line is depicted in blue with its 95% confidence interval (grey shade around the dashed line).

**Fig C. *Per*-strand mitochondrial genomic signatures of intermediate heteroplasmies in CVDs and controls**. The barplots show the aggregated frequencies of nucleotide changes (transitions and transversions) of intermediate heteroplasmic mtSNVs (HF = 10-95%) falling within the 96 nucleotide triplettes and calculated across the whole mtDNA, in each group. HTN = Hypertension, IHD = Ischemic Heart Disease, IS = Ischemic Stroke.

**Fig D.** **Distribution of mean read depths per mtDNA position** **in hypertension cases and controls**. Mean site-specific coverage for the hypertension samples and controls (WTCCC) which passed quality filtering and have at least 1 mtSNV. There was no significant difference in the per-base coverage between these two groups (Wilcoxon P-value = 0.55).

**Fig E. Frequency distribution of coefficient of variation (CV) of HF values for mtDNA variants in the hypertension (HTN) cases.** CV was calculated as the ratio between standard deviation of HF and the mean HF values calculated at each position across all samples of this study (N = 6,214 samples).

**Table A. Demographics of the three CVDs and controls.** Number of individuals reported are those with at least 1 mtSNV. HTN = Hypertension, IHD = Ischemic Heart Disease, IS = Ischemic Stroke.

**Table B. Results of the multivariate regression analysis with heteroplasmy burden and predictors in disease/controls comparisons**. In bold are P-values below the Bonferroni corrected threshold (P < 0.008, considering alpha level = 0.05 and six tests). Mt ancestry = mitochondrial ancestry (i.e. European, African, Asian); se = standard error.

**Table C. Results of the multivariate regression analysis with burden of intermediate heteroplasmies in Hypertension/controls comparison, *per* functional category.** In bold are P-values below the Bonferroni corrected threshold (P < 0.01, considering alpha level = 0.05 and five tests). Mt ancestry = mitochondrial ancestry (i.e. European, African, Asian); se = standard error.

**Table D. Results of the multivariate regression analysis with heteroplasmy burden and additional predictors in Hypertension**. Results are based on N = 1,071 hypertensive individuals with non-missing predictors. In bold is the P-value below the Bonferroni corrected threshold (P < 0.02, considering alpha level = 0.05 and two tests). Mt ancestry = mitochondrial ancestry (i.e. European, African, Asian); se = standard error; SBP = systolic blood pressure; DBP = diastolic blood pressure; Smoker = ever smoked cigarettes, cigars or pipes.

# **Supporting Materials and Methods**

## **Statistical analysis**

The number of intermediate and low heteroplasmies *per* individual was used as dependent variable in a negative binomial regression analysis to assess the additive effect of each predictor on changes in individual burden of heteroplasmies, with age, sex, mean read depth, and mitochondrial ancestry as covariates (**Table B**). Systolic and diastolic blood pressure and smoking status were included as additional cofactors in the HT analysis when possible (**Table D**). Negative binomial regression was also used to look for changes in individual heteroplasmic burden in pairwise comparisons between diseases and controls using the same covariates (**Table C**). Multivariate regression analysis between individual burden of mtSNVs and covariates was performed using a negative binomial regression model as shown in (1) and (2)

1. glm.nb(N ~ age + gender, mean read depth + ancestry)
2. glm.nb(N ~ case/control + age + gender, mean read depth + ancestry)

where N is the sum of heteroplasmies within a certain class (either intermediate or low) *per* individual. Unit measurement of age was years, gender was expressed as a binary variable, mean read depth was expressed as average number of reads mapped on mtDNA, mitochondrial ancestry was encoded as 0 for individuals with European haplogroups (H, J, K, I, T, U, V, X, W), 1 for individuals with African haplogroups (L) and 2 for individuals with Asian haplogroups (A, B, F, M, N, Y). For HT, we tested also the association with three additional covariates: systolic, diastolic pressure (expressed as mmHg) and smoking status, the latter encoded as 1 for those who ever smoked cigarettes, cigars or pipe, 0 for those who never smoked. Case/Control in (2) indicates a dummy variable encoded as 1 for CVDs and 0 for controls. The *glm.nb* R package was used and the analysis performed in R(1).

*Per* locus burden analysis was performed using SKAT-O within the R package “SKAT”(2), also incorporating the same covariates, and considering 39 mtDNA loci annotations (i.e. 13 protein coding genes, 22 tRNAs, 2rRNAs, D-LOOP and intergenic positions as one aggregated locus) and 1,657 overlapping ties of 100bp. Intergenic regions have been defined as an aggregate region of 89 positions placed between mitochondrial gene annotations. The burden test was performed separately on intermediate and low heteroplasmic mtSNVs. We performed a SKAT-O test separately on intermediate and low heteroplasmies in diseases compared to controls. Disease (equal to 1) and control status (equal to 0) was set as a dependent variable, while the occurrence of an heteroplasmic event (equal to 1, equal to 0 if no event was observed) was treated as independent predictor, together with additional covariates (age, gender, sequencing mean read depth and mitochondrial ancestry). The *skat* R package was used for this analysis and performed in R(1). We adopted a Bonferroni adjusted P-value threshold, considering an alpha level = 0.05 and the number of tests performed in each reported comparison

## **Haplogroup enrichment analysis**

We performed multinomial regression analysis to check whether European haplogroups (H, J, K, I, T, U, V, X, W) were enriched in CVDs compared to controls. For this analysis we subset on individuals with European haplogroups only, that represented the majority of each cohort and were at similar percentages across cases and controls: 95.6% of HTN, 95.5% of IHD, 94.7% of IS and 95% of controls. We used the *multinom* R package to perform the test and set the H haplogroup (the most common in Europe) as background and performed pairwise comparisons between each CVD and controls, encoding each CVD as 1 and controls as 0. None of the haplogroups was significantly enriched at P < 0.016 (considering a Bonferroni correction, alpha level = 0.05 and three tests

**Supporting Figures**

**Fig A. Distribution of mean read depths per mtDNA position.** Means have been calculated across all samples that passed quality filtering and have at least 1 mtSNV (N = 6,214, reported in **S2 Dataset**). Black dots correspond to mean read depth values, connected by solid back lines. Orange lines indicate standard deviation from the mean read depth.

**
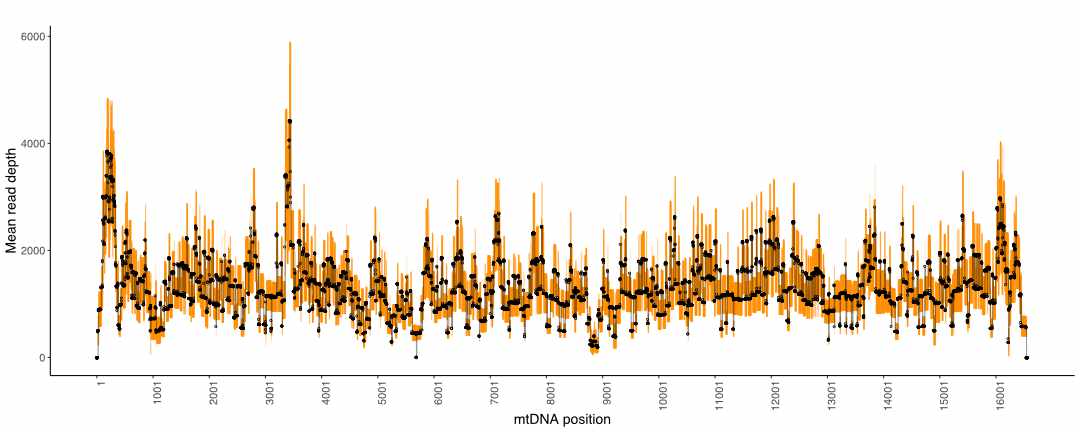
**

**Fig B. Correlation between non-synonymous heteroplasmic fractions and MutPred probability.** Shown are Spearman’ P-value and rho coefficient of correlation. The regression line is depicted in blue with its 95% confidence interval (grey shade around the dashed line).

**Fig C. *Per*-strand mitochondrial genomic signatures of low heteroplasmies and intermediate heteroplasmies in CVDs and controls.** The barplots show the 6 aggregated frequencies of nucleotide changes (transitions and transversions) of A) low heteroplasmic mtSNVs (HF = 5-10%) and B) intermediate heteroplasmic mtSNVs (HF = 10-95%) falling within the 96 nucleotide triplets and calculated across the whole mtDNA, in each group. HTN = Hypertension, IHD = Ischemic Heart Disease, IS = Ischemic Stroke.


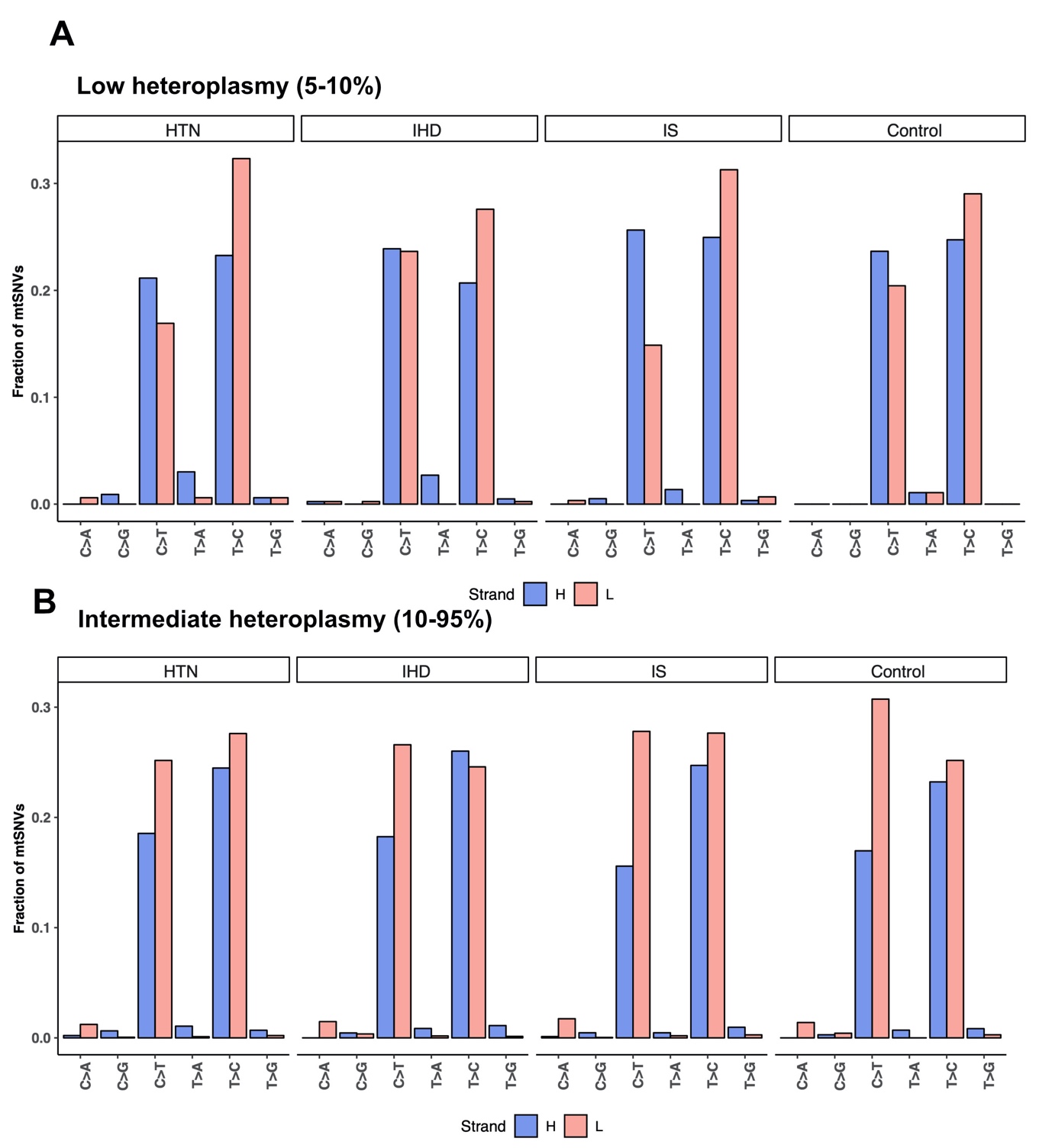


#

**Fig D.** **Distribution of mean read depths per mtDNA position** **in hypertension cases and controls**. Mean site-specific coverage for the hypertension samples and controls (WTCCC) which passed quality filtering and have at least 1 mtSNV. There was no significant difference in the per-base coverage between these two groups (Wilcoxon P-value = 0.55).

**
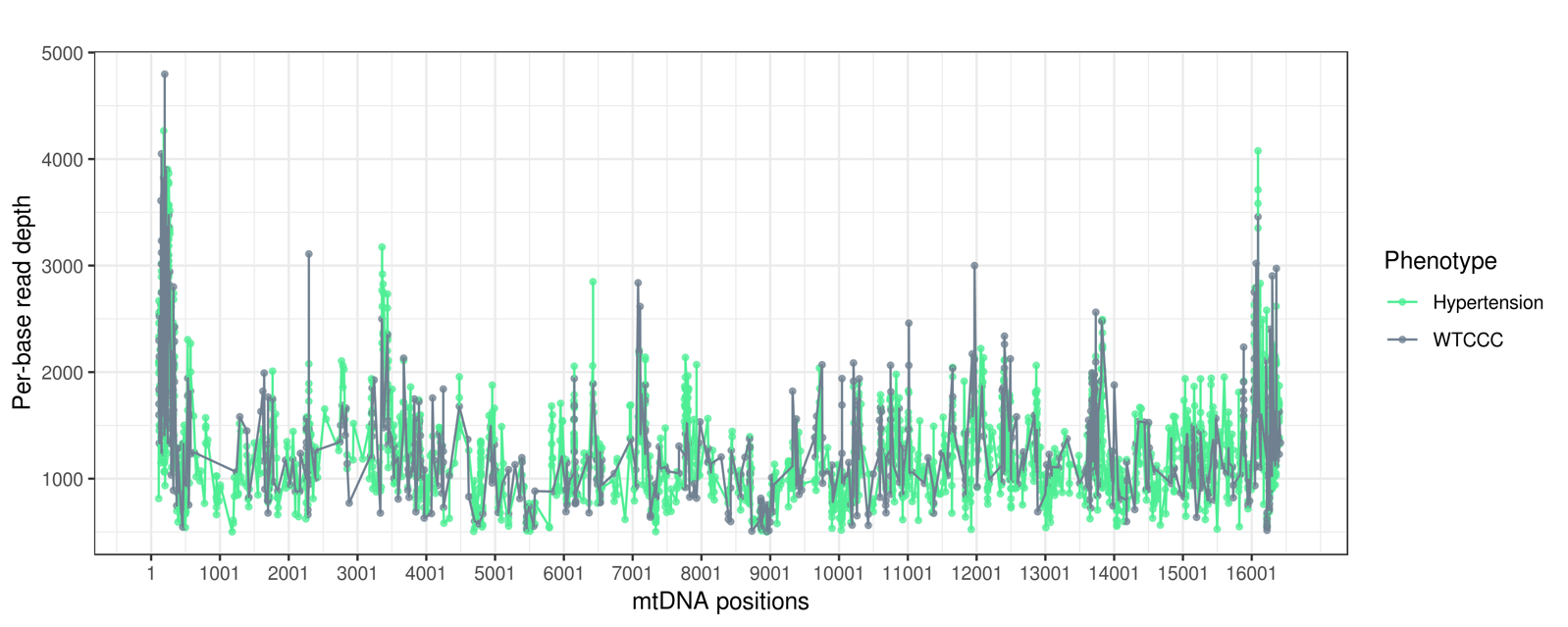
**

**Fig E. Frequency distribution of coefficient of variation (CV) of HF values for mtDNA variants in the hypertension (HTN) cases.** CV was calculated as the ratio between standard deviation of HF and the mean HF values calculated at each position across all samples of this study (N = 6,214 samples).

#
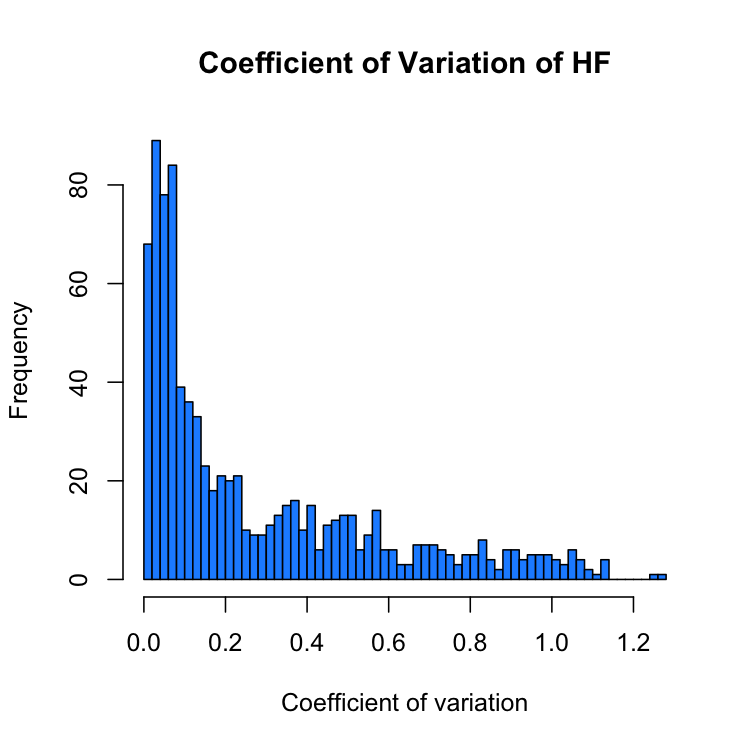


# **Supporting Tables**

**Table A. Demographics of the three CVDs and controls.**

|  |  |  | Gender | | Age (yrs) | | |
| --- | --- | --- | --- | --- | --- | --- | --- |
| **Group** | **No. individuals** | **Mean**  **read**  **depth (±sd)** | **No. Males**  **(%)** | **No. Females**  **(%)** | **1st**  **quartile** | **Median** | **3rd quartile** |
| **HTN** | 1,399 | 1047.9 (±148.6) | 579  (41%) | 820  (59%) | 50 | 57 | 64 |
| **IHD** | 1,946 | 1178.1 (±276.2) | 1,537 (79%) | 409  (21%) | 55 | 61 | 66 |
| **IS** | 2,146 | 1061.1 (±266.2) | 1,111 (52%) | 1,035  (48%) | 63 | 74 | 82 |
| **Controls** | 723 | 999.4 (±220.1) | 362  (50%) | 361  (50%) | 35 | 45 | 53 |

Number of individuals reported are those with at least 1 mtSNV. HTN = Hypertension, IHD = Ischemic Heart Disease, IS = Ischemic Stroke.

**Table B. Results of the multivariate regression analysis with heteroplasmy burden and predictors in disease/controls comparisons.**

|  |  | **predictor** | **beta** | **se** | **P-value** |
| --- | --- | --- | --- | --- | --- |
| **Intermediate heteroplasmies** | **Hypertension** | Case/Control | 0.274671 | 0.063535 | **1.54E-05** |
|  |  | Gender | 0.076791 | 0.051097 | 0.132878 |
|  |  | Age | 0.001452 | 0.002261 | 0.520831 |
|  |  | Mean read depth | 0.00052 | 0.000145 | **0.000329** |
|  |  | Mt ancestry | 0.426947 | 0.151909 | **0.004946** |
|  | **Ischemic Stroke** | Case/Control | 1.02E-01 | 7.32E-02 | 0.164 |
|  |  | Gender | 3.62E-02 | 4.53E-02 | 0.424 |
|  |  | Age | 1.97E-03 | 1.73E-03 | 0.255 |
|  |  | Mean read depth | 4.81E-04 | 8.64E-05 | **2.48E-08** |
|  |  | Mt ancestry | 5.03E-01 | 1.03E-01 | **9.51E-07** |
|  | **Ischemic Heart Disease** | Case/Control | 0.144211 | 0.072622 | 0.047057 |
|  |  | Gender | 0.042874 | 0.05435 | 0.430195 |
|  |  | Age | -0.004571 | 0.002506 | 0.068155 |
|  |  | Mean read depth | 0.000446 | 8.72E-05 | **3.19E-07** |
|  |  | Mt ancestry | 0.653525 | 0.175838 | **0.000202** |
| **Low heteroplasmies** | **Hypertension** | Phenotype | 0.364487 | 0.162538 | 0.02493 |
|  |  | Gender | -0.053471 | 0.127371 | 0.67463 |
|  |  | Age | 0.015566 | 0.005736 | **0.00666** |
|  |  | Mean read depth | 0.000188 | 0.000368 | 0.60993 |
|  |  | Mt ancestry | 0.162142 | 0.422541 | 0.70118 |
|  | **Ischemic Stroke** | Case/Control | 1.35E-01 | 1.64E-01 | 0.409 |
|  |  | Gender | 1.70E-02 | 9.19E-02 | 0.853 |
|  |  | Age | 2.04E-02 | 3.74E-03 | **4.69E-08** |
|  |  | Mean read depth | 5.17E-05 | 1.75E-04 | 0.768 |
|  |  | Mt ancestry | -2.09E-01 | 2.85E-01 | 0.464 |
|  | **Ischemic Heart Disease** | Case/Control | 0.296396 | 0.179756 | 0.0992 |
|  |  | Gender | -0.16131 | 0.128699 | 0.2101 |
|  |  | Age | 0.010918 | 0.006225 | 0.0795 |
|  |  | Mean read depth | 0.000244 | 0.00021 | 0.2462 |
|  |  | Mt ancestry | -0.185366 | 0.620855 | 0.7653 |

In bold are P-values below the Bonferroni corrected threshold (P < 0.008, considering alpha level = 0.05 and six tests). Mt ancestry = mitochondrial ancestry (i.e. European, African, Asian); se = standard error.

**Table C. Results of the multivariate regression analysis with burden of intermediate heteroplasmies in Hypertension/controls comparison, *per* functional category.**

| **Functional category** | **predictor** | **beta** | **se** | **P-value** |
| --- | --- | --- | --- | --- |
| **D-loop** | Case/Control | 0.21 | 0.10 | 0.0399 |
|  | Gender | 0.00 | 0.00 | 0.7723 |
|  | Age | 0.12 | 0.08 | 0.1601 |
|  | Mean read depth | 0.00 | 0.00 | 0.1356 |
|  | Mt ancestry | 0.60 | 0.21 | 0.0042 |
| **Non-synonymous** | Case/Control | 0.28 | 0.08 | **0.000562** |
|  | Gender | 0.00 | 0.00 | 0.252609 |
|  | Age | 0.10 | 0.07 | 0.134935 |
|  | Mean read depth | 0.00 | 0.00 | **9.36E-05** |
|  | Mt ancestry | 0.32 | 0.20 | 0.105951 |
| **Synonymous** | Case/Control | 0.33 | 0.32 | 0.3039 |
|  | Gender | 0.00 | 0.01 | 0.9086 |
|  | Age | -0.26 | 0.26 | 0.3143 |
|  | Mean read depth | 0.00 | 0.00 | 0.0151 |
|  | Mt ancestry | 0.47 | 0.64 | 0.4602 |
| **tRNA** | Case/Control | 0.68 | 0.32 | 0.0308 |
|  | Gender | 0.00 | 0.01 | 0.8438 |
|  | Age | 0.44 | 0.24 | 0.0673 |
|  | Mean read depth | 0.00 | 0.00 | 0.8528 |
|  | Mt ancestry | -19.32 | 11540.00 | 0.9987 |
| **rRNA** | Case/Control | 0.47 | 0.19 | 0.0126 |
|  | Gender | 0.00 | 0.01 | 0.6415 |
|  | Age | -0.25 | 0.15 | 0.0848 |
|  | Mean read depth | 0.00 | 0.00 | 0.7313 |
|  | Mt ancestry | 0.24 | 0.42 | 0.5726 |

In bold are P-values below the Bonferroni corrected threshold (P < 0.01, considering alpha level = 0.05 and five tests). Mt ancestry = mitochondrial ancestry (i.e. European, African, Asian); se = standard error.

**Table D. Results of the multivariate regression analysis with heteroplasmy burden and additional predictors in Hypertension**.

|  | **predictor** | **beta** | **se** | **P-value** |
| --- | --- | --- | --- | --- |
| **Intermediate heteroplasmies** | Age | 0.0085375 | 0.0033988 | **0.0120** |
|  | Gender | 0.0370158 | 0.0702721 | 0.5984 |
|  | Mean read depth | 0.0004319 | 0.0002270 | 0.0571 |
|  | Mt ancestry | 0.1798007 | 0.2702634 | 0.5059 |
|  | SBP | -0.0044424 | 0.0024541 | 0.0703 |
|  | DBP | 0.0082544 | 0.0044550 | 0.0639 |
|  | Smoker | -0.0820770 | 0.0965826 | 0.3954 |
| **Low**  **heteroplasmies** | Age | 0.0271100 | 0.0083055 | **0.001098** |
|  | Gender | 0.1296683 | 0.1673813 | 0.438524 |
|  | Mean read depth | 0.0004536 | 0.0005418 | 0.402484 |
|  | Mt ancestry | 0.1867845 | 0.6309733 | 0.767210 |
|  | SBP | -0.0077338 | 0.0058293 | 0.184603 |
|  | DBP | 0.0141790 | 0.0105446 | 0.178733 |
|  | Smoker | -0.3292369 | 0.2454946 | 0.179883 |

Results are based on N = 1,071 hypertensive individuals with non-missing predictors. In bold is the P-value below the Bonferroni corrected threshold (P < 0.02, considering alpha level = 0.05 and two tests). Mt ancestry = mitochondrial ancestry (i.e. European, African, Asian); se = standard error; SBP = systolic blood pressure; DBP = diastolic blood pressure; Smoker = ever smoked cigarettes, cigars or pipes.

# **Supporting References**

1. R Core Team (2014). R: A language and environment for statistical computing. R Foundation for Statistical Computing, Vienna, Austria.

2. S. Lee, *et al.*, Optimal Unified Approach for Rare-Variant Association Testing with Application to Small-Sample Case-Control Whole-Exome Sequencing Studies. *The American Journal of Human Genetics* **91**, 224–237 (2012).
